# Unpacking amphotericin B toxicity: a critical review of amphotericin B aggregation and its link to toxicity and antifungal activity

**DOI:** 10.1007/s00204-026-04420-0

**Published:** 2026-05-20

**Authors:** Chandra Harshita Chavali, Evelyne Deplazes

**Affiliations:** School of Chemistry and Molecular Biosciences, University of Queensland, St Lucia, QLD 4072 Australia

**Keywords:** Amphotericin B, Polyene drugs, Antifungal drugs, Aggregation, Toxicity, Haemolysis, Drug formulation

## Abstract

Amphotericin B (AmB) is a polyene antifungal that, despite its severe dose-limiting toxicity, is the most effective drug to treating invasive fungal infections. The drug’s toxicity arises from its poor selectivity for ergosterol over cholesterol and its tendency to aggregate. Understanding the relationship between toxicity and aggregation is challenging due to the drug’s complex mechanism of action, yet imperative to improve the safety profiles of existing and new polyene-based antifungal drugs. In this review, we critically examine and summarise studies that investigate the aggregation of AmB and its relationship to toxicity. As the aggregation behaviour of AmB is highly sensitive to environmental conditions, we provide an in-depth analysis of sample preparation, characterisation of aggregates and control experiments. While data from haemolysis, animal, and cell-based studies consistently show that oligomeric AmB is more toxic than larger aggregates, findings on the toxicity of monomeric AmB inconsistent. Our collated data underscores the need for careful consideration of sample preparation and proper use of vehicle controls when studying the complex relationship between the supramolecular organisation of AmB and its biological effects.

## Introduction

Amphotericin B (AmB) is a polyene macrolide naturally produced by the soil bacteria *Streptomyces nodosus*. AmB was discovered in 1956 (Berteau and Maffrand 1955; Gold and Mindlin 1956) and soon after was approved as the first effective treatment for invasive fungal infections (IFIs) due to its broad-spectrum, fungicidal activity. Even after more than 65 years of clinical use, acquired resistance to AmB remains rare. Thus, despite its severe, dose-limiting toxicity, AmB remains an important frontline drug for treating life-threatening IFIs, including those caused by *Candida auris*, *Cryptococcus neoformans*, and *Aspergillus fumigatus* (Kaminski 2014).

The toxicity of AmB is directly linked to its mechanism of action. Despite decades of research characterising the complex mechanism of action and aggregation of AmB, decoupling antifungal activity and toxicity has proven challenging. *Only recently, *Maji et al. (2023)* reported the first AmB derivative that shows reduced toxicity without losing antifungal activity. The toxicity of AmB is also affected by its intrinsic aggregation properties. Studying the role of aggregation in the mechanism and off-target effects of AmB is challenging* because aggregation of AmB is highly sensitive to environmental conditions and methods of sample preparation. It is also difficult to characterise the aggregation state of AmB in cellular environments and is usually inferred from experiments in controlled solution environment, *which assumes that the insights gained from and these experiments and sample preparation protocols transfer to samples prepared for cell-based experiments.* In this review, we critically analyse and summarise studies reporting in vivo and in vitro toxicity data on AmB in its different aggregation states. Our paper begins with a brief overview of AmB, and its mechanism and a review of techniques used to characterise the aggregation states of AmB. This is followed by an in-depth review of studies reporting the effect of aggregation on the in vivo and in vitro antifungal activity, haemolytic activity, and in vivo and in vitro toxicity in mammalian systems. To the best of our knowledge, this is the first review that collates information on aggregation and pharmacological activity while also providing a critical comparison of sample preparation, an aspect that can result in apparent contradictions of findings. While there has been extensive research into using various drug delivery or conjugation systems (e.g. emulsions, nanoparticles or suspensions) to reduce the toxicity of AmB, many of these studies generally do not explicitly focus on the role of aggregation and are thus not included in our review.

## AmB structure, formulations and mechanisms of action

**Fig. 1:**
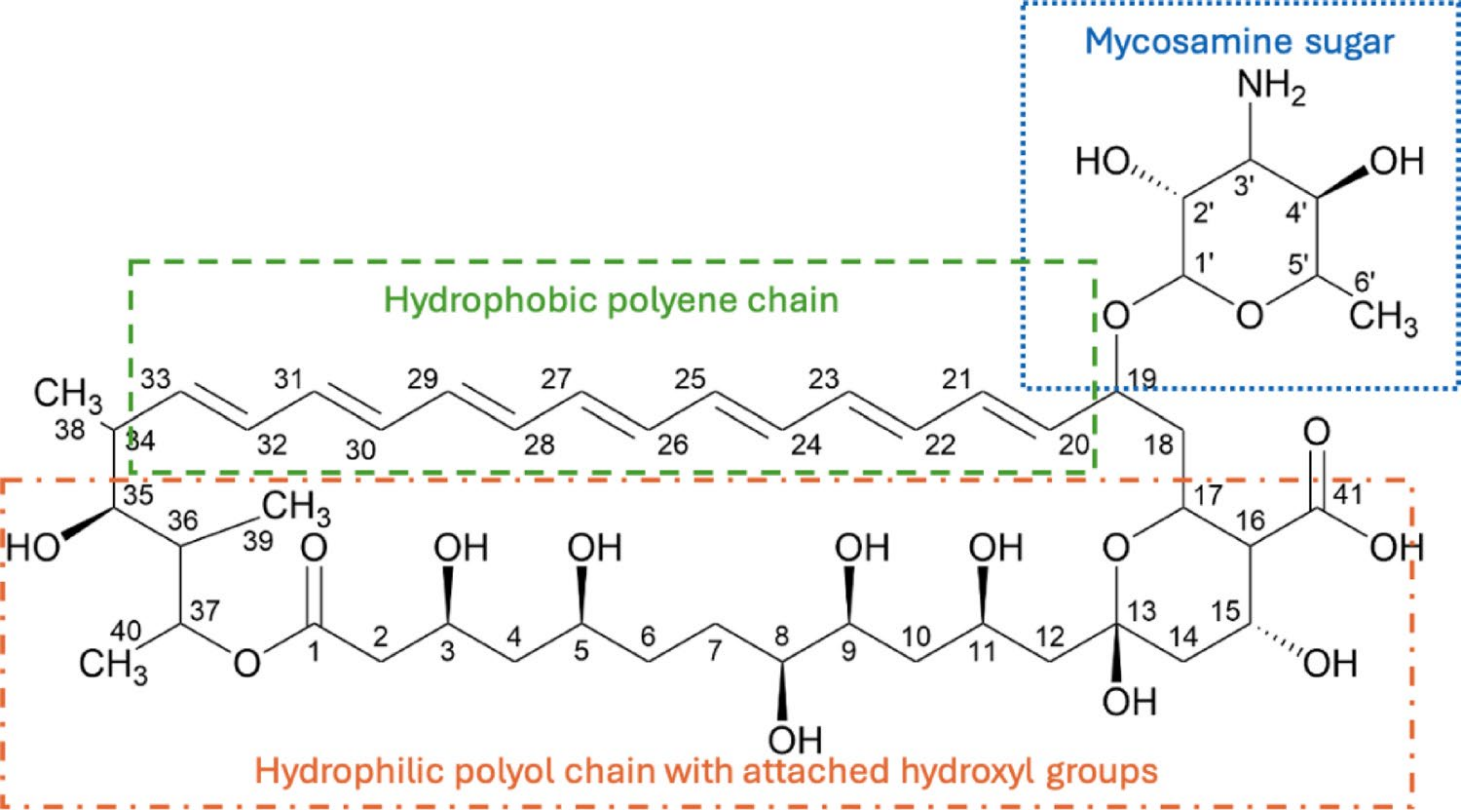
Chemical structure of AmB with its polyol, polyene and mycosamine moieties indicated

The AmB molecule is comprised of three distinct moieties, the polyene subunit spanning C20–C33, the polyol subunit spanning C3–C16 and the lactone ring with a mycosamine group at C19 (Fig. 1). The arrangement of these three moieties is responsible for the physicochemical characteristics of AmB, resulting from distinct polar and nonpolar regions and the presence of ionisable carboxyl and amine groups. This amphiphilic and zwitterionic structure, combined with an asymmetrical distribution of hydrophobic and hydrophilic moieties, renders it poorly soluble in both aqueous and many organic solvents. At physiological pH AmB is charge neutral (Kaminski 2014).

AmB interacts with the sterols present in cell membranes. Due to the high structural similarity of ergosterol and cholesterol, AmB shows only moderate selectivity for ergosterol found in fungal cells over cholesterol in mammalian cells. The affinity of AmB for ergosterol is approximately 10 × higher than for cholesterol, based on surface plasmon resonance and UV–vis spectroscopy binding assays (Mouri et al. 2008; Readio and Bittman 1982; Witzke and Bittman 1984). This lack of binding affinity and thus selectivity is the main source of toxicity of AmB in human cells. In addition to this direct interaction, the concentration-dependent aggregation state of AmB affects the selectivity and specificity. Langmuir experiments revealed that in its monomeric form, AmB shows a higher penetration into ergosterol-containing monolayers compared to cholesterol-containing monolayers, while in its aggregate form, this selectivity is reversed (Miñones et al. 2005). The increased affinity of AmB for ergosterol over cholesterol is thought to be mediated by the more rigid structure of ergosterol compared to cholesterol, which has fewer double bonds than ergosterol. Specifically, molecular dynamics simulations suggested that the rigidity of ergosterol results in increased van der Waals interactions and hydrogen bonds with AmB, alongside more favourable entropic contributions (Neumann et al. 2010, 2009).

AmB exerts its antifungal activity via a complex and multi-facetted mechanism that involves both membrane-mediated AmB-sterol interactions that cause a loss of sterol and increase in permeability in the plasma cell membrane, and cell-internal effects that result in the production of reactive oxygen species (Kaminski 2014). The different mechanisms likely exist alongside each other and depend on the concentration and aggregation state of AmB and sterol content of the cell.

**Fig. 2:**
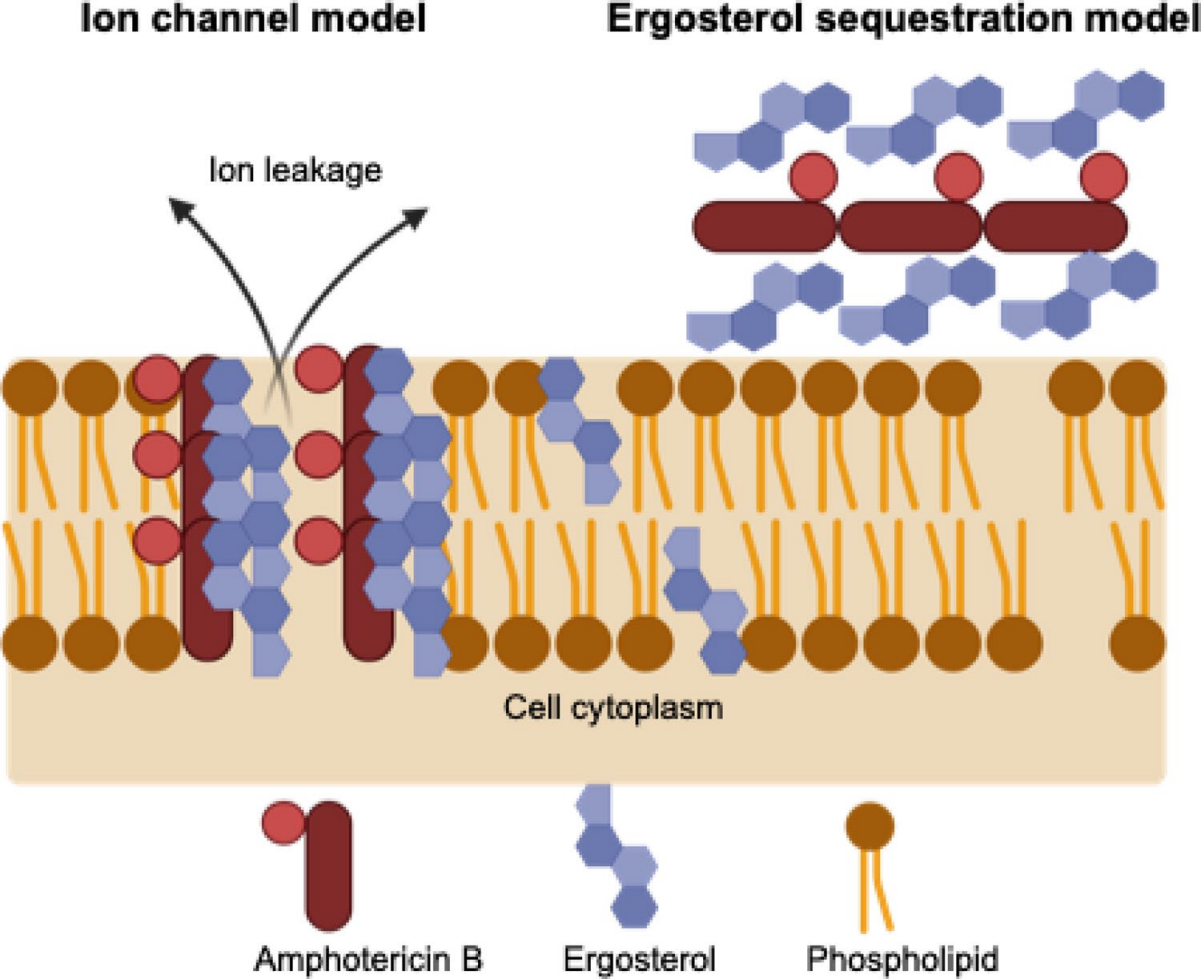
Models for the membrane-mediated interactions of AmB with fungal membranes: ion channel and ergosterol sequestration and sterol sponge models

The ion channel model is the oldest and most studied mechanism of AmB. This model proposes that AmB molecules form a pore in the fungal plasma membrane (Fig. 2). The model is based on a series of electrophysiological and biochemical studies from the 1960s and 1970s demonstrating that AmB increases the permeability of phospholipid bilayers and membranes to ions and small molecules in a voltage-independent mechanism and that this effect depends on the presence of sterols (Anderson and Lehrer 1975; Andreoli 1974; Andreoli and Monahan 1968; Bolard et al. 1991; De Kruijff and Demel 1974; Finkelstein and Holz 1973a, 1973b). This loss of electrolytes and disruption of ion homeostasis leads to cell death. While the presence of ion channels was inferred from electrophysiology experiments, in particular single-channel recordings (Anderson and Lehrer 1975; Finkelstein and Holz 1973b), the small size and membrane-embedded nature of the proposed AmB-sterol complex made structural characterisation challenging. More recently, solid-state NMR and molecular dynamics simulations were used to determine the structure of AmB ion channels in ergosterol-containing phospholipid bilayers. *This model suggests that seven AmB molecules (or AmB units) form a 10 Å-wide pore, with the polyol subunits facing inwards and the polyene subunits facing outwards *(Umegawa et al. 2022)*.*

For decades, the ion channel model was considered the sole mechanism of AmB-ergosterol interaction and thus the main mediator of the drug’s antifungal activity. This view was challenged in a series of recent landmark studies by Anderson et al. (2014); Gray et al. (2012) which revealed two new mechanisms of actions: sequestration and the sterol sponge model. The authors used a series of AmB derivatives that retain selective binding to ergosterol but cannot form ion channels to show that AmB ergosterol sequestration is the primary mode of fungicidal activity and that ion channel formation is a secondary, complementary mechanism that increases rate of killing (Gray et al. 2012). In a subsequent study, the authors used solid-state NMR and transmission electron microscopy to reveal that AmB forms large extra-membranous aggregates that are more closely associated with water than lipids. Further analysis confirmed that these aggregates actively extract ergosterol from membranes, reinforcing the validity of these non-pore-forming mechanisms of action. *The presence of extra-membranous aggregates was also demonstrated by *(Grela et al. 2019)* using fluorescence lifetime imaging in live yeast cells.* Ergosterol is critical for maintaining cell membrane stability, endocytosis, stabilisation of proteins and acts as a signalling molecule for cell internal processes. Thus, AmB-induced ergosterol sequestration causes membrane destabilisation and likely interferes with various processes vital for yeast cell physiology, ultimately leading to cell death independent of ion channel formation. Sterol sponge and sequestration are thought to work in tandem with the ion channel formation to produce the fungicidal effect of AmB (Cavassin et al. 2021; Gray et al. 2012; Palacios et al. 2011). A key difference between the mechanisms lies in the aggregation state of AmB; while ion-channel formation requires AmB to be in its monomeric form, sequestration and sterol sponge formation can occur with AmB in its monomeric or aggregated form.

This NMR structure of the sterol-sponge formed the basis of the pivotal development of a new AmB derivative that selectively extracts ergosterol over cholesterol (Maji et al. (2023). Rather than increasing the binding affinity of AmB for ergosterol this new form of AmB, Am-2-19, selectivity increases the kinetics of ergosterol sequestration resulting in significantly reduced host toxicity while retaining the broad-spectrum antifungal effects of AmB.

## Aggregation of AmB

The poor solubility of AmB requires the compound to be solubilised for intravenous administration. The earliest and still used formulation is AmB-deoxycholate (AmB-D), also known by its brand name Fungizone. Deoxycholate is a bile salt that acts as a detergent and improves the solubility and stability of AmB monomers. Until recently, decades of research to develop less toxic AmB derivatives failed to decouple the antifungal activity and toxicity. In an effort to indirectly reduce toxicity, numerous lipid formulations or emulsions were proposed. Rather than changing the AmB-sterol interaction, these formulations aim to improve the drug’s therapeutic index of AmB by increasing the selective delivery of AmB to fungal cells over mammalian cells. Such formulations include amphotericin B lipid complex, liposomal amphotericin B, and amphotericin B colloidal dispersion (Hamill 2013; Wang et al. 2021). Approved by the FDA in 1997, AmBiosome is a liposomal formulation of AmB with increased circulation time and lower toxicity, resulting in a more effective treatment. However, the high cost of AmBiosome means it is not accessible to many low-income countries where IFIs are most prevalent (Liu et al. 2023) and mortality rates are higher than in more advanced health care settings. Each of these formulations exhibits different toxicity profiles, which may arise from the differences in the supramolecular states of AmB within the formulations. Understanding the supramolecular chemistry of AmB is thus still a critical factor to improving the drug’s therapeutic index and guide the development of other polyene-based antifungal drugs.

**Fig. 3:**
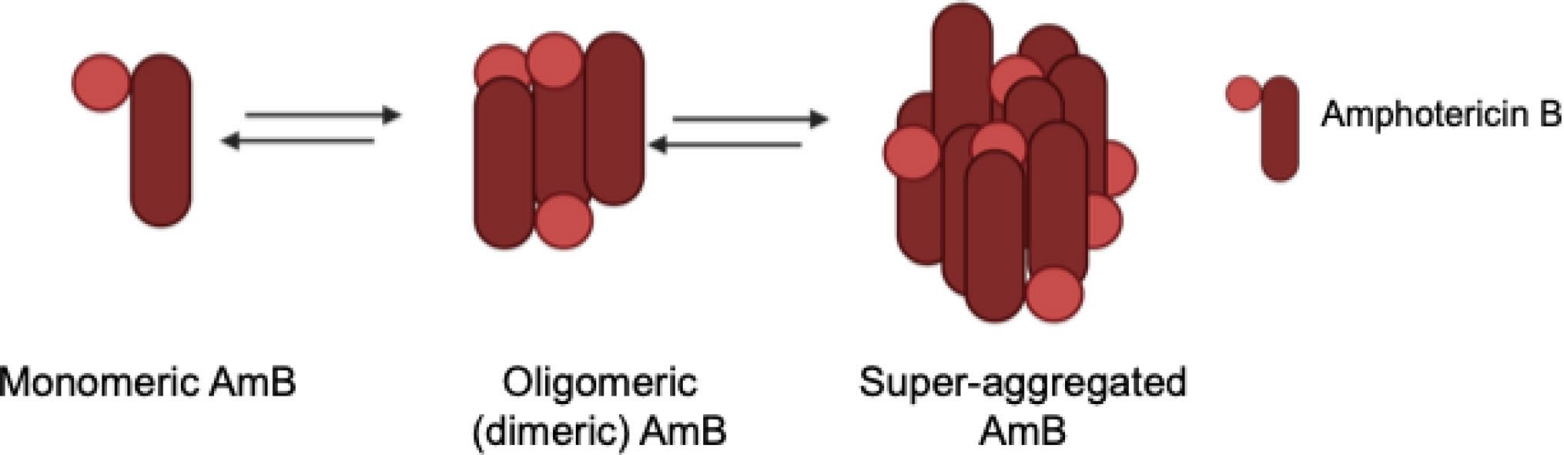
Representations of the aggregation states of AmB: monomeric, oligomeric, and super-aggregate AmB

Due to its amphipathic character, AmB is poorly soluble in aqueous solutions (or any other polar solvent). Thus, under physiologically relevant conditions, AmB predominantly exists in various aggregation states with only a fraction of monomers present (Fig. 3). The concentration at which AmB starts to aggregate is referred to as the critical aggregate concentration (CAC). The aggregation states are generally classified into three groups: monomers, oligomers (previously referred to as dimers) and super-aggregates. These states exist in an equilibrium which is sensitive to AmB concentration, temperature, solvent polarity, and ionic strength of solvent environment and the method used to prepare the solution (e.g. duration of agitation, centrifugation or heat treatment). Maintaining AmB in its monomeric form without any oligomers or aggregates forming requires solubilisation in organic solvents such as DMSO. In one of the first studies on aggregation, Mazerski et al. (1990) proposed a stepwise aggregation model where AmB monomers initially interact with each other in aqueous media to form dimers or other small aggregates called oligomers (Fig. 3). These oligomers further interact to form larger aggregates, with aggregate growth occurring by the addition of oligomers rather than monomers. This phenomenon was explained by the structural incompatibility of the AmB monomer’s lipophilic part with the water-exposed, hydrophilic surface of aggregates.

## Techniques to characterise AmB

As the different aggregation states exhibit distinct pharmacological and toxicological profiles, establishing a relationship between aggregation and activity relies on accurate and reliable characterisation of the monomeric, oligomeric and super-aggregate forms of AmB. This is mostly achieved using UV–vis spectroscopy, circular dichroism (CD) spectroscopy or dynamic light scattering (DLS). It is important to note that the aggregation state of AmB is usually determined in an ex-vivo, controlled solution environment, and the presence of different aggregate forms in cellular environments is then implied from these results.

**Fig. 4:**
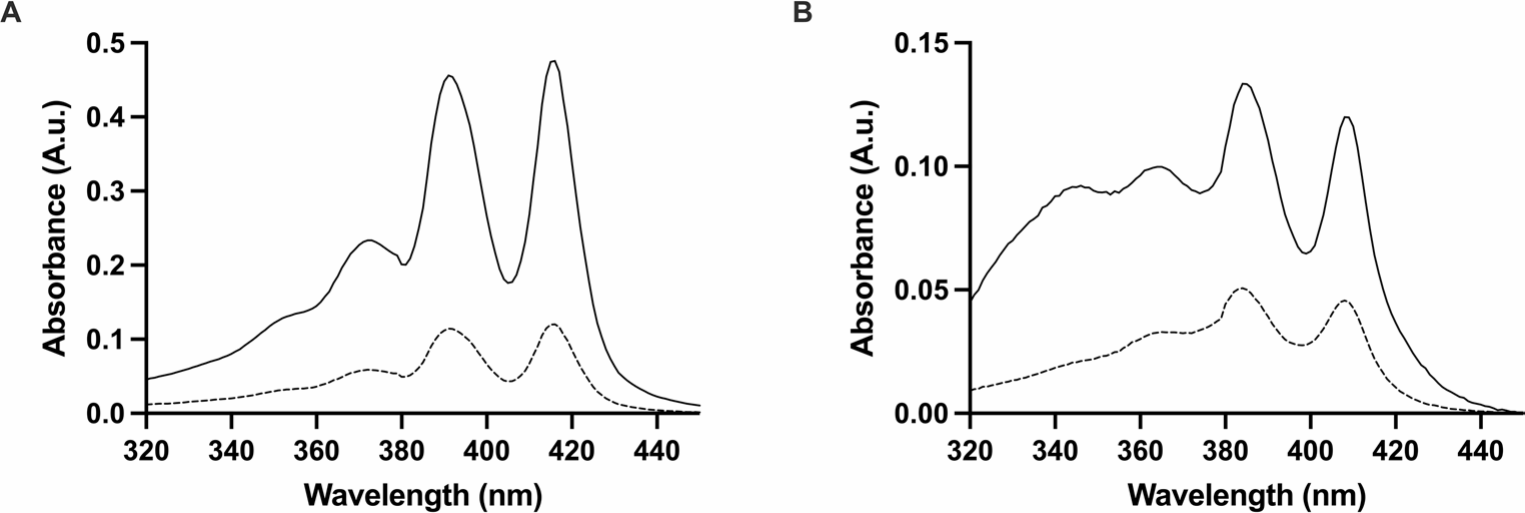
UV–vis spectra of AmB in DMSO (**A**) and tris buffer (**B**) at 1 µM (dotted line) and 4 µM (solid line). The spectrum for monomeric AmB (**A**) is characterised by two high-intensity peaks at 406–409 nm and 420 nm and low-intensity peaks near 360 nm and 380 nm. For AmB oligomers or aggregates, the peaks at 360 nm and 380 nm are shifted hypsochromically, and the 340 nm peak broadens and is much higher in intensity

UV–vis spectroscopy is the most used technique to characterise AmB’s degree of aggregation. Monomeric AmB exhibits two high-intensity peaks at 406–409 nm and 420 nm, along with two low-intensity peaks near 360 nm and 380 nm (Fig. 4A). In oligomeric AmB, the 360 nm and 380 nm peaks undergo a hypsochromic shift, in oligomeric AmB while aggregates show a prominent increase in absorbance for the 340 nm peak (Fig. 4B) (Barwicz, et al. 1992, and Gaboriau et al. 1997a, b).

The relative intensity of these characteristic absorbance peaks is a reliable method to determine whether AmB monomers or aggregates dominate. One method to quantify the degree of AmB aggregation is plotting the ratio of absorbance at 328 nm and 409 nm. Reported values for this ratio range from 0.003 to 0.25 for monomers, 1.35–2 for oligomers, and 0.48 for super-aggregates (Barwicz et al. 1992; Fernandez-Garcia et al. 2022). The peak at 409 nm has also been used to calculate the CAC by plotting the molar absorption coefficient vs the reciprocal of concentration (Aramwit et al. 2000; Grela et al. 2023). When comparing data from different studies, it is critical to consider the method of preparation as this affects the position of the aggregate peak. For example, heat-induced super-aggregates exhibit a hypsochromic shift compared to oligomers, with the dominant peak now at 320 nm. In contrast, centrifugation-prepared super-aggregates display a distinct spectral pattern, with a newly emerging peak at 358 nm, followed by secondary peaks at 370 nm, 392 nm, and 420 nm (Fernandez-Garcia et al. 2022; Gaboriau et al. 1997a; Tancrede et al. 1990). UV–vis spectroscopy is frequently combined with DLS to confirm CAC values.

CD spectroscopy is another technique regularly used to characterise monomeric, oligomeric and super-aggregated forms of AmB. Monomeric AmB lacks any significant dichroic doublet (Gaboriau et al. 1997a; Legrand et al. 1992b; Sanchez-Brunete et al. 2004). In contrast, aggregated forms of AmB show a more distinct CD doublet. The key difference between oligomeric and super-aggregated AmB is the hypsochromic shift exhibited by super-aggregated AmB compared to the oligomeric AmB spectra (Rochelle do Vale Morais et al. 2018; Sanchez-Brunete et al. 2004). CD spectroscopy provides a comparative analysis of AmB aggregation states but cannot be used to obtain quantitative data such as the monomer-to-aggregate ratio or CAC due to the absence of a spectra for the monomeric form. Therefore, it is often used in combination with UV–vis spectroscopy.

*While CD spectroscopy in itself cannot provide structural information, work by *Starzyk et al. (2014)* showed that detailed analysis of vibrational bands in the CD spectra can be used to identify dimers. When combined with structural information from molecular dynamics simulations and fluorescence lifetime imaging (FLIM) distinguish between parallel and antiparallel dimers. Analysis of time evolution of CD spectra showed that these dimers can further associate into tetramers. In the same study, the authors extended the use of FLIM and fluorescence correlation spectroscopy to show that these tetramers also exist in membrane environments. In a similar study, *Gruszecki et al. (2009)* used FLIM to show differences in AmB aggregation for ergosterol and cholesterol- containing membranes, suggesting that ergosterol might increase the presence of monomers, thus linking the dynamic monomer–dimer-aggregate equilibrium to selectivity. The ability to use FLIM to detect AmB aggregates was also used to demonstrate that AmB binds to cell walls and cell membranes in the form of small aggregates *(Grela et al. 2019)*. Compared to CD and UV–vis spectroscopy, FLIM imaging enables simultaneous observation of molecular organisation, including distinguishing monomeric from oligomeric and aggregate forms based on fluorescence lifetime, and localisation of the drug in the cell.*

*It is important to note that spectroscopic techniques commonly used to infer the aggregation state of AmB, such as UV–Vis and CD, are susceptible to confounding effects. Work from the *(Gagoś and Czernel 2014)* group has shown that spectral changes often attributed to aggregation can also arise from oxidation of AmB. These findings underscore an important methodological limitation, indicating that aggregation-related interpretations, particularly in earlier spectroscopic studies conducted without adequate protection against oxidation, should be approached with caution and may, in some cases, require reinterpretation or repetition.*

DLS is complementary to spectroscopy methods as it provides information on the size of particles and aggregates, which cannot be obtained from UV–vis or CD spectroscopy. Monomeric AmB has a small radius of approximately 1 nm, whereas oligomeric forms exhibit significantly larger diameters, ranging from 11 to 49 nm (Espada et al. 2008; Fernandez-Garcia et al. 2022). The size of super-aggregated AmB varies depends on the preparation method. Heat-induced aggregation results in particle sizes exceeding 1000 nm, while super-aggregates formed through centrifugation have a comparatively smaller radius of approximately 400 nm (Espada et al. 2008; Fernandez-Garcia et al. 2022). By monitoring changes in the scattering profiles as AmB transitions from monomeric form to aggregates, DLS can also be used to determine the CAC For example, Barwicz et al. (1992) and Gaboriau et al. (1997a) used DLS to measure the CAC of AmB by identifying the sudden change in the DLS signal intensity. Their findings also indicated that the transition of oligomeric AmB to super-aggregates was marked by a further increase in the DLS signal, confirming that the super-aggregated form represents a higher-order aggregation state than the oligomeric form.

## Effects of aggregation state on AmB’s haemolytic activity in human RBCs

The haemolytic activity of AmB on human erythrocytes is commonly used to assess the toxicity of various formulations and preparation methods of AmB. Early studies of AmB formulations and their varying selectivity toward cholesterol and ergosterol led researchers to hypothesise that the differences in toxicity are due to the variation in the aggregation state of AmB (Barwicz et al. 1992; Legrand et al. 1992a). To address this hypothesis, a series of studies compared the haemolytic activity of pure AmB, Fungizone (the commercially available form of AmB-D) or AmB-D prepared in the lab using various methods to control aggregation, with a particular focus on comparing the monomeric state to that of oligomers and larger aggregates (Table 1). In most cases, the haemolytic assays are combined with experiments to characterise the aggregation states of the different samples using techniques such as UV–vis and CD-spectroscopy, DLS (Espada et al. 2008; Gaboriau et al. 1997b; Legrand et al. 1992a) and more recently NMR and TEM (Fernandez-Garcia et al. 2022; Serrano et al. 2013).

**Table 1:** Summary of studies investigating the effect of AmB aggregation state on its haemolytic activity in human red blood cells

References	Compounds tested	Characterisation of AmB monomers and aggregates	Haemolysis experiments		Methods of reporting effect and overall findings
			AmB conc. tested	Conditions of experiments	
Legrand et al. (1992a, b)	Pure AmB^1^	*CD*^*2*^* and UV–vis spectroscopy* Monomers — 0.1 mM AmB dissolved in DMSO or DMF diluted in PBS to 1 – 5 µM Oligomeric aggregates —10 mM and 4.2 mM AmB dissolved in DMSO and DMF, respectively, diluted in PBS to obtain 1 – 5 µM	1 – 10 µM (1 – 10 µg/mL)	*Human RBC* *K*^*+*^* leakage detected using FAAS*^*2*^ 2 × 10^8^ cells/mL 1 h incubation at 37 °C Control – RBC without AmB	*%K*^*+*^* leakage caused by 3 µM AmB* Monomers 0.1 mM AmB-DMSO stock – 30% mM AmB-DMF stock – 50% Oligomeric aggregates 10 mM AmB-DMSO stock – 65% 4.2 mM AmB-DMF stock – 60% → aggregates showed a higher K + leakage compared to monomers
Gaboriau et al. (1997a, b)	AmB-D^4^; AmB dissolved in phosphate salts (to mimic Fungizone)	*UV–vis spectroscopy and DLS*^*5*^ No monomers tested Oligomeric aggregates – AmB-D dissolved in ultrapure water / PBS Super-aggregates – Heated aggregates- AmB-D dissolved in ultrapure heated at 70 °C for 20 min	0 – 10 µM (0 – 13.3 ug/mL)	*Human RBC* *K*^*+*^* leakage using FAAS* 4 × 10^7^ cells/mL used 1 h incubation at 37 °C Control – RBC without AmB *Haemoglobin leakage detected by UV–vis* Same prep as K^+^ leakage Reference for 100% leakage – RBC with ultrapure water	*Concentration required for 50% leakage K*^*+*^* leakage* AmB-D – 0.3 mM Heated AmB-D – 1 mM *Concentration required for 100% Haemolysis* AmB-D – 10 µM Heated AmB-D > 50 µM → oligomers showed a more haemolysis at a than heated super-aggregates
Espada et al. (2008)	AmB pure, Fungizone, AmB-D; AmB dissolved in phosphate salts (to mimic Fungizone)	*UV–vis spectroscopy and DLS* Monomers prepared using γ cyclodextrin + pure AmB in ultrapure water Oligomeric aggregates – AmB-D dissolved in ultrapure water Super-aggregates. i) poly-aggregate – water-insoluble, prepared similar to oligomeric aggregates but centrifuged for several cycles at 4500xg and supernatant used for testing. ii) Heated fungizone—Fungizone dissolved in ultrapure water heated at 70 °C for 20 min	0 – 200 µg/mL	*Human RBC* *Haemoglobin leakage detected by UV–vis* 7 × 10^9^ cells/mL 1 h incubation at 37 °C Reference for 100% leakage – RBC with ultrapure water	*% Haemolysis at 100 µg/mL of AmB* cyclodextrin + AmB – 33.1 (Same as γ-cyclodextrin control without AMB) AmB-D – 5.07% AmB-poly-aggregate – 0.04% Heated Fungizone – 3.17% → monomeric AmB showed highest haemolysis, followed by oligomeric AmB, heated fungizone and AmB-poly-aggregate. However, cyclodextrin exhibited similar haemolysis value suggesting that the toxicity could be due to cyclodextrin rather than AmB itself
Serrano et al. (2013)	AmB pure, Fungizone, AmB-D-AmB dissolved in phosphate salts (to mimic Fungizone)	*UV–vis spectroscopy, DLS, SEM*^*6*^*, and TEM*^*7*^ No monomers Oligomeric aggregates – AmB-D dissolved in ultrapure water Super-aggregates. (i) (poly-aggregate – water-insoluble) prepared similar to AmB-D but centrifuged for several cycles at 4500xg and supernatant used for testing, (ii) Heated fungizone – Fungizone dissolved in ultrapure heated at 70 °C for 20 min	200 µg/mL	*Human RBC* *Haemoglobin leakage detected by UV–vis* 3.5 × 10^9^ cells/mL 1 h incubation at 37 °C Reference for 100% leakage – RBC with ultrapure water	*% Haemolysis at 200 µg/mL after 5 h* AmB-D – 80% AmB-poly-aggregate – 2% Heated Fungizone – 30% → Oligomeric AmB-D showed the highest haemolysis at measured conc. while AmB-poly-aggregated form shows the lowest haemolysis
Silva-Filho et al. (2012)	AmB-D-AmB dissolved in phosphate salts (to mimic Fungizone)	*UV–vis spectroscopy* No monomers Oligomeric aggregates – AmB-D dissolved in ultrapure water Super-aggregates – Heated aggregates – AmB-D dissolved in ultrapure heated at 70 °C for 20 min	0.05 – 50 µg/mL	*Human RBC* *K*^*+*^* leakage using FAAS* 5 × 10^7^ cells/mL used 1 h incubation at 37 °C Control- RBC without AmB *Haemoglobin leakage detected by UV–vis* Same prep as K^+^ leakage Reference for 100% leakage – RBC with ultrapure water	*Concentration required for 50% leakage K*^*+*^* leakage* AmB-D – 0.5 µg/mL Heated AmB-D – 0.5 µg/mL *Concentration required for 100% Haemolysis* AmB-D – 5 µg/mL Heated AmB-D > 50 µg/mL → oligomers show a higher haemolysis than heated super-aggregates
Fernández-García et al., (2022)	AmB pure, AmB-D-AmB dissolved in phosphate salts (to mimic Fungizone)	*CD, UV–vis spectroscopy, DLS, NMR*^*8*^*, TEM, SEM* Monomers prepared using cyclodextrin + pure AmB in ultrapure water Oligomeric aggregates – AmB-D dissolved in ultrapure water Super-aggregates—(poly-aggregate – water-insoluble) prepared similar to AmB-D but centrifuged for several cycles at 4500xg and supernatant used for testing	1.69 – 270 µM (2.26 – 361 ug/mL)	*Human RBC* *Haemoglobin leakage detected by UV–vis* Same prep as K^+^ leakage Reference for 100% leakage – RBC with Triton	*Concentration required for 50% Haemolysis* cyclodextrin + AmB **–** 23 µM AmB-D – 120 µM AmB-poly-aggregate – 438 µM → monomeric AmB showed highest haemolysis, followed by oligomeric AmB and super-aggregated AmB

^1^AmB- Amphotericin B, ^2^CD spectroscopy- Circular dichroism spectroscopy, ^3^FAAS- Flame atomic absorption spectroscopy, ^4^AmB-D- Amphotericin Deoxycholate, ^5^DLS- Dynamic light scattering, ^6^SEM- Scanning electron microscopy, ^7^TEM- Transmission electron microscopy, ^8^NMR- Nuclear magnetic resonance

AmB monomers were usually prepared by dissolving pure AmB in organic solvents such as DMSO or DMF (Legrand et al. 1992a). An alternative preparation involves dissolving pure AmB with cyclodextrin in ultrapure water (Espada et al. 2008; Fernandez-Garcia et al. 2022). In most studies, the presence of monomeric AmB was confirmed immediately after sample preparation. It is important to note that the 1-h incubation of the sample with RBCs in media likely results in a further increase in aggregation. As the sample was not re-analysed after incubation it is not known to what extent monomers were still present during the haemolysis experiments.

Oligomeric AmB was usually prepared by one of three techniques. The first method involved directly using commercially available Fungizone, which contains AmB with sodium deoxycholate and sodium phosphate. The second method involved using commercially formulated AmB-D powder, which was then dissolved in buffer. The third approach was to prepare AmB-D in the laboratory by dissolving pure AmB with sodium deoxycholate in sodium phosphate buffer at pH 12.0 to mimic the composition of Fungizone. These solutions were then used to prepare super-aggregates of AmB by either centrifuging or heating the sample to 70 ºC for 20 min (Table 1).

Haemolysis was quantified either by measuring potassium (K⁺) leakage by flame atomic absorption spectroscopy (FAAS) or by measuring haemoglobin leakage by UV–vis spectroscopy. Both assays measure leakage of cell-internal content and are thus an indirect measure of RBC membrane disruption. Data is generally reported as % haemolysis for the tested range of AmB concentrations or as the concentration required to induce a specific level of haemolysis. Comparison of haemolytic activity between studies is often complicated by variations in protocols, in particular, what solution is used to define 100% haemolysis. Another potential limitation of some studies is the lack of a vehicle control, which makes it challenging to distinguish if the toxicity was solely due to AmB or by the solvents/excipients used, e.g. cyclodextrin (Fernandez-Garcia et al. 2022).

Multiple studies have reported varying levels of haemoglobin leakage for the different aggregation states of AmB, making it challenging to directly compare the values (Espada et al. 2008; Fernandez-Garcia et al. 2022; Gaboriau et al. 1997a). Espada et al. tested 100 µg/mL of AmB and found that monomers induced the highest leakage at 33.1%, followed by oligomers at 5% leakage, while aggregates exhibited the lowest leakage, ranging from 0.04 to 3.17%. However, the study noted that the haemoglobin leakage observed with monomeric AmB (prepared using cyclodextrin + AmB) was identical to that of its vehicle control (cyclodextrin alone). This raised concerns that the observed toxicity might be attributed to the vehicle rather than AmB itself, leading the authors to conclude that oligomeric AmB is likely the most toxic form.

A study by Fernández-García et al. found monomers to be more toxic than oligomers or aggregates. Specifically, monomers induced 50% leakage at 23 µM (21.25 µg/mL), followed by oligomers at 120 µM (110.8 µg/mL) and super-aggregates at 428 µM (404 µg/mL). While these findings suggest monomers are the most toxic, the study did not include a vehicle control to assess the potential toxicity of cyclodextrin, making direct comparisons between the three aggregation states more challenging.

Several other studies (Gaboriau et al. 1997b; Serrano et al. 2013; Silva-Filho et al. 2012) compared oligomeric AmB and super-aggregate AmB. Despite the variations between the results due to the different preparation methods in the studies, it was consistently shown that oligomeric AmB is more toxic, inducing 100% leakage at 5–10 µM, whereas super-aggregated AmB required over 50 µM to cause similar leakage.

## Effects of the aggregation state of AmB on its in vivo toxicity in mammalian animal models

Various studies used mammalian animal models to understand the relationship between the aggregation state of AmB to its in vivo toxicity (Table 2). Similar to the haemolysis studies, various solvents/excipients or preparation methods to obtain samples with AmB in different aggregation states, as assessed by DLS, UV–vis or CD spectroscopy.

**Table 2:** Summary of studies investigating the effect of AmB aggregation state on its in vivo toxicity of AmB in mammals

References	Compounds tested	Characterisation of AmB monomers and aggregates	Animal experiments		Methods of reporting effect and overall findings
			AmB dose tested	Conditions of experiments	
Barwicz et al. (1992)	AmB-D, Fungizone	*UV–vis spectroscopy and DLS* Monomers – AmB + LS^1^ AmB-D (1:42 ratio of AmB to Deoxycholate to maintain AmB in its monomeric form) Oligomeric aggregates – Fungizone (1:1.8 ratio of AmB to Deoxycholate)	1.7 – 5.1 mg/kg	*Mice* *Mortality over 7 days* Healthy mice were injected with AmB, and mortality monitored over 7 days	*% survival on day 7* Fungizone – 6% AmB-D – 28% AmB-LS – 38% →oligomeric AmB showed higher toxicity than monomeric AmB in mice
Kwong et al. (2001)	Fungizone, Heated Fungizone	*Characterisation of AmB was not done* No characterisation. Authors referred to previous studies showing that Fungizone at the tested concentrations is oligomeric and heated-Fungizone forms super-aggregates	1 mg/kg	*Rabbit* *Renal toxicity assessed by serum creatinine levels* Healthy rabbits were injected with AmB, and serum creatine levels were measured 10 h post-treatment	*% change of creatinine from baseline after 10 h* Fungizone – 51.7% Heated Fungizone – 13.2% → oligomeric AmB showed higher toxicity than heated super-aggregates
Sivak et al. (2004)	Fungizone, Heated Fungizone	*Characterisation of AmB was not done* No characterisation. Authors referred to previous studies showing that Fungizone at the tested concentrations is oligomeric and heated-Fungizone forms super-aggregates	1 mg/kg	*Rats* *Renal toxicity assessed by serum creatinine levels* Rats infected with *Aspergillus fumigatus* were injected with AmB and serum creatine levels were measured 4 days post-treatment	*% change of creatinine from baseline after 4 days* Fungizone – 1.6% Heated Fungizone – 16.7% → oligomeric AmB showed higher toxicity than heated super-aggregates
Sánchez-Brunete et al., (2004)	pure AmB, AmB-D; AmB dissolved in phosphate salts (to mimic Fungizone)	*CD and UV–vis spectroscopy* Monomers prepared using cyclodextrin + pure AmB in ultrapure water Oligomeric aggregates – AmB-D dissolved in ultrapure water Super-aggregates—poly-aggregate – water-insoluble, prepared similar to oligomeric aggregates but centrifuged for several cycles at 4500 × g and supernatant used for testing	2 – 20 mg/kg	*Hamsters* *Mortality after 2 days* Hamsters infected with *Leishamania* were injected with AmB and mortality recorded after 48 h	*Concentration of AmB that caused 100% mortality* cyclodextrin + AmB – 5 mg/kg AmB-D – 20 mg/kg AmB-poly-aggregate – > 20 mg/kg → monomeric AmB showed the highest toxicity, followed by oligomeric AmB and poly-aggregate AmB
Espada et al. (2008)	Pure AmB, Fungizone, AmB-D; AmB dissolved in phosphate salts (to mimic Fungizone)	*UV–vis spectroscopy and DLS* Monomers prepared using cyclodextrin + AmB Oligomeric aggregates – AmB-D dissolved in ultrapure water Super-aggregates—(poly-aggregate – water-insoluble) prepared similar to oligomeric aggregates but centrifuged for several cycles at 4500xg and supernatant used for testing	2 – 40 mg/kg	*Mice* *Survival after 2 days* Healthy mice injected with AmB, and mortality recorded after 48 h	*Concentration of AmB that caused 100% mortality* cyclodextrin + AmB – 15 mg/kg AmB-D – 10 mg/kg AmB-poly-aggregate – > 40 mg/kg → oligomeric AmB showed the highest toxicity, followed by monomeric AmB and poly-aggregate AmB

^1^LS, Lauryl sulphate

All studies used rodents, with mice and rats being the most common. Two main testing approaches were used: (1) administering AmB to healthy animals to assess acute toxicity and (2) administering AmB to infected rodents testing for mortality/serum creatine levels. The toxicity was generally assessed as either mortality rates or by organ toxicity as determined by a biomarker (e.g. creatin levels as an indicator of renal toxicity).

Kwong et al. (2001) and Sivak et al. (2004) compared acute renal toxicity of Fungizone to that of heated Fungizone administered to healthy rabbits and mice infected with *Aspergillus fumigatus*, respectively, by monitoring the changes in serum creatine levels from baseline either 10 h or 4 days post-treatment. However, neither study characterised the aggregation state of AmB tested. Instead, the authors referred to previous studies demonstrating that Fungizone forms oligomers while heated Fungizone forms super-aggregates. Despite differences in experimental conditions, both studies found that heated Fungizone is less toxic. This is based on changes in serum creatine values of -16.7% vs 1.6% (Sanchez-Brunete et al. 2004) and 51.7% vs 13.2% (Kwong et al. 2001) for Fungizone and heated Fungizone.

Barwicz et al. (1992), Sanchez-Brunete et al. (2004) and Espada et al. (2008) compared the toxicity of monomeric AmB with its oligomeric and/or super-aggregate forms. The monomers were prepared by dissolving pure AmB with lauryl sulphate (Barwicz et al. 1992) or cyclodextrin (Espada et al. 2008; Sanchez-Brunete et al. 2004) in ultrapure water, while oligomeric AmB was prepared by dissolving Fungizone (Barwicz et al. 1992) or AmB-D (Espada et al. 2008; Sanchez-Brunete et al. 2004) in ultrapure water. Super-aggregates were prepared by heating the solution of AMB-D to 70ºC. The aggregation states were characterised using CD and UV–vis spectroscopy. Both Barwicz et al. (1992) and Espada et al. (2008) reported survival of healthy mice injected with the samples and found that oligomeric AmB was less toxic than monomeric AmB. Espada et al. (2008) reported mouse mortality at 15 mg/kg for monomeric AmB and 10 mg/kg for oligomeric, while super-aggregated AmB exhibited the lowest toxicity, with mortality occurring at > 40 mg/kg. Similarly, Barwicz et al. (1992), reported a 6% survival of mice treated with oligomeric AmB compared to a 28–38% survival of mice treated with monomeric AmB. In contrast, Sanchez-Brunete et al. (2004) who tested infected hamsters found monomers to be more toxic than oligomers. The variations between monomeric and oligomeric forms may be influenced by differences in experimental conditions, including animal models and infection status. Nevertheless, the three studies agreed in their findings that aggregates showed the lowest toxicity.

## Effects of the aggregation state of AmB on its toxicity against mammalian cell lines

Compared to haemolysis and in-vivo studies there is less research assessing the cytotoxic effects of different AmB aggregates in mammalian cell lines (Table 3). In the two studies identified in this review, cell viability was evaluated using MTT (Gaboriau et al. 1997b) and MTS assays (Bartlett et al. 2004; Egito et al. 2003) in HT29 and LLC-PK cell lines, respectively. In both studies, the aggregation state of AmB was not directly characterised. Both studies tested Fungizone (previously reported to be oligomeric) and heated Fungizone (previously reported to be a super-aggregate). The results indicated that Fungizone exhibited higher toxicity, with an IC_50_ of 6 µg/mL in HT29 cells and 20 µg/mL in LLC-PK cells, whereas heated Fungizone demonstrated reduced toxicity, with IC_50_ values of 40 µg/mL and 50 µg/mL, respectively. While these findings suggest that the aggregation state significantly influences the cytotoxicity of AmB, the lack of direct characterisation of the samples limits the ability to precisely correlate toxicity with specific aggregate forms or compare it to the effect of AmB monomers.

**Table 3:** Summary of studies investigating the effect of AmB aggregation state on its in vivo toxicity of AmB in mammalian cell lines

References	Compounds tested	Characterisation of AmB monomers and aggregates	Mammalian cell line experiments		Methods of reporting effect and overall findings
			AmB dose tested	Conditions of experiments	
Gaboriau et al. (1997b)	AmB-D^4^; AmB dissolved in phosphate salts (to mimic Fungizone); heated AmB-D	*UV–vis spectroscopy and DLS*^*5*^ No monomers tested Oligomeric aggregates – AmB-D dissolved in ultrapure water / PBS Super-aggregates – Heated aggregates- AmB-D dissolved in ultrapure heated at 70 °C for 20 min	1 × 10^–8^–2 × 10^–5^ M (0.013–13 µg/mL)	*HT29 cell line (colon cancer cells)* *Cell viability measured using an MTT assay* HT29 cells were treated with AMB, incubated for 15 h and evaluated for cell viability with an MTT assay	*Concentration causing 50% cell death* Fungizone – 6 µM Heated Fungizone – 40 µM → oligomeric AmB shows higher toxicity than heated super-aggregates
Bartlett et al. (2004)	Fungizone, Heated Fungizone	*Characterisation of AmB was not done* No characterisation. Authors referred to previous studies showing that Fungizone at the tested concentrations is oligomeric and heated-Fungizone forms super-aggregates	0.3–16 µg/mL	*LLC-PK*_*1*_* cell line (pig kidney cells)* *Cell viability measured using an MTS assay* LLC-PK_1_ cells were treated with AMB, incubated for 18 h and evaluated for cell viability with an MTS assay	*Concentration causing 50% cell death* Fungizone – 20 µg/mL Heated Fungizone –50 µg/mL → oligomeric AmB shows higher toxicity than heated super-aggregates

## Summary of findings from studies on the effect of aggregation on AmB’s toxicity in mammalian systems

Data from haemolysis, animal, and cell-based studies consistently indicate that oligomeric AmB exhibits the highest toxicity, followed by its super-aggregated form. This trend is observed across both in vitro and in vivo studies, regardless of differences in aggregation preparation methods or toxicity assessment techniques.

However, the monomeric form of AmB presents conflicting results. Some studies report it as the most toxic form, while others suggest it is less toxic than oligomeric AmB. This inconsistency likely arises from two key factors. First, maintaining AmB in its monomeric state is challenging, particularly in aqueous environments where spontaneous aggregation occurs at very low drug concentrations. Thus, there is no certainty that AmB remains monomeric throughout the testing. In addition, the size of oligomers and aggregates is sensitive to the method of sample preparation, such as method and duration of agitation, centrifugation as well as solution conditions (pH, temperature, ionic strengths). This prevents a direct comparison between data sets from different studies. Second, the excipients and solvents required to stabilise monomeric AmB often possess inherent toxicity. Since not all studies included appropriate vehicle controls, it is possible that toxicity attributed to monomeric AmB was, at least in part, due to these excipients rather than AmB itself. Consequently, some of the reported toxicity data may not accurately reflect the true toxicity of monomeric AmB.

## Effect of AmB aggregation state on its in vitro antifungal activity

To assess the impact of aggregation states of AmB on its antifungal activity, several studies have compared the ability of monomeric, oligomeric and aggregate AmB to reduce the growth of *Candida* and *Cryptococcus* species (Table 4) (Bartlett et al. 2004; Fernandez-Garcia et al. 2022; Gaboriau et al. 1997b; van Etten et al. 2000). These studies involved culturing fungal cells to the desired confluency, incubating them with AmB, and measuring subsequent cell viability. Studies comparing AmB oligomers to heated-AmB super-aggregates found no significant difference in antifungal activity between the two forms of AmB (Bartlett et al. 2004; Gaboriau et al. 1997b; van Etten et al. 2000). However, a reduction in activity was observed when poly-aggregates generated through centrifugation rather than heat treatment were compared to oligomers (Fernandez-Garcia et al. 2022). Additionally, (Rochelle do Vale Morais et al. 2018) investigated the effects of AmB aggregation on *Leishmania* species and similarly found no significant variation in activity across different aggregation states. These findings suggest that while super-aggregates may reduce the toxicity of AmB, they do not appear to alter its antifungal interactions.

**Table 4:** Effect of AmB aggregation state on its in vitro antifungal activity

References	Compounds tested	Characterisation of AmB monomers and aggregates	MIC experiments		Methods of reporting effect and overall findings
			Conc. of AmB tested	Conditions of experiments	
Gaboriau et al. (1997a, b)	AmB-D^4^; AmB dissolved in phosphate salts (to mimic Fungizone)	*UV–vis spectroscopy and DLS* No monomers tested Oligomeric aggregates – AmB-D dissolved in ultrapure water / PBS Super-aggregates – Heated aggregates- AmB-D dissolved in ultrapure heated at 70 °C for 20 min	0.02 – 5 µM (0.026 – 6.69 µg/mL)	*Candida albicans* *C. albicans* cells grown to 1 × 10^6^ CFU/mL were incubated with AmB • for 20 min and tested for growth density • overnight and tested for IC_50_	*Decrease in cell density at 5 µM AmB* AmB-D – 47% Heated AmB-D – 45% **IC**_**90**_ AmB-D – 2 mM Heated AmB-D – 2 mM → No significant difference between the antifungal effects of oligomeric AmB-D and super-aggregates from heated AmB-D
van Etten Els, van Vianen, Roovers, & Frederik, (2000)	Fungizone, Heated Fungizone	*Cryo-electron microscopy* No monomers tested Oligomeric aggregates – Fungizone dissolved in ultrapure water / PBS Super-aggregates – Heated aggregates- Fungizone dissolved in ultrapure heated at 70 °C for 20 min	0.025 – 1.6 mg/L	*Candida albicans* *C. albicans* cells grown to 1.3 × 10^7^ CFU/mL were incubated with AmB for 6 h and tested for growth	*IC*_*90*_ Fungizone – 0.1 mg/L Heated Fungizone – 0.1 mg/L → No significant difference between the antifungal effects of oligomeric AmB-D and super-aggregates from heated AmB-D
Bartlett et al. (2004)	Fungizone, heated Fungizone	*Characterisation of AmB was not done* No characterisation. Authors referred to previous studies showing that Fungizone at the tested concentrations is oligomeric and heated-Fungizone forms super-aggregates	0.03–16 µg/mL	*Cryptococcus neoformans* *Cryptococcus neoformans* cells grown to 1 × 10^4^ CFU/mL were incubated with AmB for 48 h and tested for growth	*IC*_*50*_ Fungizone – 0.125 – 1 µg/mL Heated Fungizone – 0.125 – 1 µg/mL → No significant difference between the antifungal effects of Fungizone and heated Fungizone
Rochelle do Vale Morais et al. (2018)	Fungizone, heated Fungizone	*UV–vis and CD spectroscopy* No monomers tested Oligomeric aggregates – Fungizone dissolved in ultrapure water / PBS Super-aggregates – Heated aggregates- Fungizone dissolved in ultrapure heated at 70 °C for 20 min	0.39–100 µg/mL	*Leishmania donovani* *Leishmania donovani* parasites grown to 5 × 10^6^ CFU/mL were incubated with AmB for 72 h and tested for growth	*IC*_*50*_ Fungizone – 0.05 µM Heated Fungizone – 0.05 µM → No significant difference between the antifungal effects of Fungizone and heated Fungizone
Fernández-García et al., (2022)	Pure AmB, AmB-D-AmB dissolved in phosphate salts (to mimic Fungizone)	*CD, UV–vis spectroscopy, DLS, NMR*^*9*^*, TEM, SEM* Monomers prepared using cyclodextrin + pure AmB in ultrapure water Oligomeric aggregates – AmB-D dissolved in ultrapure water Super-aggregates—(poly-aggregate – water-insoluble) prepared similar to AmB-D but centrifuged for several cycles at 4500 × g and supernatant used for testing	1.69–270 µM (2–361 µg/mL)	*Candida spp.* *Candida spp.* cells grown to 0.2 absorbance (600 nm) were incubated with AmB for 48 h and tested for growth	*IC*_*50*_ *C. albicans* cyclodextrin + AmB **–** 5.6 nM AmB-D – 12.4 nM AmB-poly-aggregate – 443.5 nM → monomeric AmB showed highest antifungal activity, followed by oligomeric AmB and super-aggregated AmB

## Effect of AmB aggregation state on its in vivo antimicrobial activity

In vivo testing of the antimicrobial activity of the different aggregation states of AmB was conducted in either mice, rats, or hamsters (Table 5). The animals were first infected with either fungal or leishmanial species, followed by the administration of either oligomeric or super-aggregated forms of AmB. antimicrobial activity was assessed by measuring the parasite burden in the animal’s post-infection.

**Table 5:** Effect of AmB aggregation state on its in vivo antifungal activity

References	Compounds tested	Characterisation of AmB monomers and aggregates	Animal experiments		Findings
			Dosage of AmB tested	Conditions of experiments	
Petit et al. (1999)	Fungizone, Heated-Fungizone	*Characterisation of AmB was not done* No characterisation. Authors referred to previous studies showing that Fungizone at the tested concentrations is oligomeric and heated-Fungizone forms super-aggregates	0.04 – 1 mg/kg	*Mice* Mice infected with *Leishmania donovani* were injected with AmB and tested for ED_90_ 7 days post infection	*ED*_*50*_* of infection* Fungizone – 0.361 mg/kg Heated Fungizone – 0.144 mg/kg →heated super-aggregated AmB showed a higher antileishmanial activity than oligomeric AmB
van Etten Els et al., (2000)	Fungizone, Heated-Fungizone	*Cryo-electron microscopy* No monomers tested Oligomeric aggregates – Fungizone dissolved in ultrapure water / PBS Super-aggregates – Heated aggregates- Fungizone dissolved in ultrapure heated at 70 °C for 20 min	0.025 – 1.6 mg/L	*Mice* Mice infected with *Candida albicans* were injected with AmB and tested for ED_90_ 7 days post infection	*Log*_*10*_* CFU of Candida albicans in mice kidney at 0.8 mg/kg of AmB* Fungizone – 5.1 Heated Fungizone – 5.0 →No significant difference between the antifungal activity of oligomeric AmB-D and heated super-aggregate AmB-D
Sánchez-Brunete et al., (2004)	AmB dissolved in phosphate salts (to mimic Fungizone), Heated-AmB-D	*CD and UV–vis spectroscopy* Oligomeric aggregates – AmB-D dissolved in ultrapure water Super-aggregates — (poly-aggregate – water-insoluble) prepared similar to oligomeric aggregates but centrifuged for several cycles at 4500xg and supernatant used for testing	2 mg/kg	*Hamsters* Hamsters infected with *Leishmania infantum* were injected with AmB and tested for toxicity through parasite burden 150 days post infection	*% reduction of parasite burden* AmB-D – 64.3% Poly-aggregate- Amb-D – 81.8% →heated-AmB-D showed a higher antileishmanial activity than fungizone
Sivak et al. (2004)	Fungizone, Heated-Fungizone	*Characterisation of AmB was not done* No characterisation. Authors referred to previous studies showing that Fungizone at the tested concentrations is oligomeric and heated-Fungizone forms super-aggregates	1 mg/kg	*Rats* Rats infected with *Aspergillus fumigatus* were injected with AmB and tested for toxicity through CFU/mL 4 days post infection	*CFU/0.5 g of homogenised tissue* Fungizone – 1354 Heated-Fungizone – 867 →No significant difference between the antifungal activity of oligomeric AmB and heated super-aggregate AmB
Rochelle do Vale Morais et al. (2018)	Fungizone, heated-Fungizone	*UV–vis and CD spectroscopy* No monomers tested Oligomeric aggregates – Fungizone dissolved in ultrapure water / PBS Super-aggregates – Heated aggregates- Fungizone dissolved in ultrapure heated at 70 °C for 20 min	1 mg/kg	*Mice* Mice infected with *Leishmania donovani* were injected with AmB and tested for toxicity through parasite burden 7 days post treatment	*% reduction of parasite burden* Fungizone – 72% Heated-Fungizone – 78% →No significant difference between the antifungal activity of oligomeric AmB and heated super-aggregate AmB

Petit et al. (1999), Sanchez-Brunete et al. (2004), and Rochelle do Vale Morais et al. (2018) investigated the antileishmanial activity of AmB in mice and hamsters. While Petit et al. (1999) and Sanchez-Brunete et al. (2004) found that super-aggregated AmB demonstrated higher antifungal activity in vivo compared to oligomeric AmB, Rochelle do Vale Morais et al. (2018) observed no significant difference between the two forms. van Etten et al. (2000) and Sivak et al. (2004) examined the antifungal activity of AmB in rats infected with *Candida albicans* and *Aspergillus fumigatus,* respectively and found no significant difference in antifungal activity between oligomeric and super-aggregated AmB.

These findings suggest that the aggregation state does not impact the antifungal activity of AmB. However, the inconsistencies in the leishmanial studies highlight the need for further research to determine whether factors such as host species or infection models influence the effectiveness of AmB.

Overall, the studies suggest that the in vitro and in vivo antifungal activity of AmB remains consistent despite its aggregation state. While the heating of AmB reduces its toxicity, it does not affect its antifungal activity.

## Conclusion

Our review is the first in-depth analysis of studies that relate the aggregation state of AmB to its pharmacological activity and in vivo and in vitro toxicity. Data from haemolysis, animal, and cell-based studies consistently show that oligomeric AmB is more toxic than super-aggregates. In contrast, reports on monomeric AmB are inconsistent, largely because this form is difficult to maintain and often aggregates during testing. Toxicity assessments are further complicated by preparation-dependent differences in aggregate size and by the use of excipients needed to stabilise monomers, some of which are themselves toxic. As a result, part of the toxicity attributed to monomeric AmB in some studies may reflect the effects of these vehicles rather than the drug itself. In contrast to host toxicity, the antifungal activity of AmB is a lot less sensitive to the drug’s aggregation state. Our collated data underscores the need to consider the method preparation when assessing the complex relationship between the supramolecular organisation of AmB.

## Data Availability

The authors declare that the data supporting the fi ndings of this study are available within the paper.
